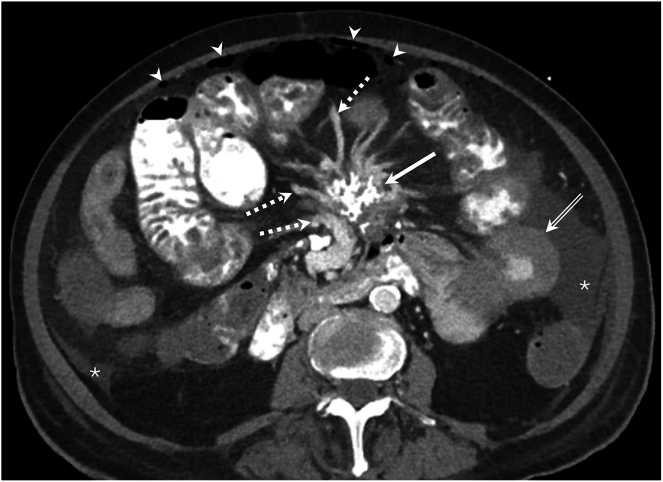# Scarred From the Inside: A Case of Idiopathic Sclerosing Mesenteritis

**DOI:** 10.1016/j.gastha.2025.100759

**Published:** 2025-07-24

**Authors:** Miguel E. Gomez, Michael L. Wells

**Affiliations:** 1 Mayo Clinic Rochester Division of Internal Medicine, Rochester, Minnesota; 2 Mayo Clinic Rochester Division of Radiology, Rochester, Minnesota

This is an 80-year-old female Jehovah’s witness who was admitted for progressive dyspnea in the setting of acute on chronic normocytic anemia. Her labs were notable for anemia with a hemoglobin of 7.3 g/dL (decreased from 8.1 g/dL 3 months prior). Computed tomography abdomen pelvis demonstrated calcifications of the central bowel mesentery concerning for sclerosing mesenteritis. Gastroenterology performed an upper endoscopy, which demonstrated Los Angeles grade C esophagitis and Cameron erosions without active hemorrhage. Infectious and autoimmune workups were unremarkable. Surgical biopsy was deferred. She was treated with IV iron sucrose and erythropoietin and discharged on proton-pump inhibitor therapy.

She was readmitted soon after for worsening anemia and progressive abdominal swelling. She was initiated on diuretic therapy for rapidly accumulating ascites and prednisone and tamoxifen for sclerosing mesenteritis. Repeat computed tomography abdomen pelvis (Figure) showed free intraperitoneal air (arrow heads), ascites in the paracolic gutters (asterix), ischemia in the jejunum in the ascending colon with progressive jejunal thickening (double arrow), dilated central mesenteric veins (dotted arrows) and a mass with irregular calcification in the central mesentery (arrow). Bowel perforation was diagnosed based on the presence of intraperitoneal free air and peritoneal fluid analysis. She transitioned to comfort care and passed away.

**Figure 1:**